# Differential Expression Analysis for RNA-Seq Data

**DOI:** 10.5402/2012/817508

**Published:** 2012-09-20

**Authors:** Rashi Gupta, Isha Dewan, Richa Bharti, Alok Bhattacharya

**Affiliations:** ^1^School of Computational and Integrative Sciences, JNU, New Delhi 110067, India; ^2^CorrZ Technosolutions Pvt. Ltd., Noida 201304, India; ^3^Indian Statistical Institute, New Delhi 110016, India; ^4^School of Life Sciences, JNU, New Delhi 110067, India

## Abstract

RNA-Seq is increasingly being used for gene expression profiling. In this approach, next-generation sequencing (NGS) platforms are used for sequencing. Due to highly parallel nature, millions of reads are generated in a short time and at low cost. Therefore analysis of the data is a major challenge and development of statistical and computational methods is essential for drawing meaningful conclusions from this huge data. In here, we assessed three different types of normalization (transcript parts per million, trimmed mean of M values, quantile normalization) and evaluated if normalized data reduces technical variability across replicates. In addition, we also proposed two novel methods for detecting differentially expressed genes between two biological conditions: (i) likelihood ratio method, and (ii) Bayesian method. Our proposed methods for finding differentially expressed genes were tested on three real datasets. Our methods performed at least as well as, and often better than, the existing methods for analysis of differential expression.

## 1. Introduction

 One of the recent methods for gene expression profiling is RNA-Seq. An advantage of RNA-Seq over other gene expression profiling technologies is that it allows a comprehensive assay that does not require probes for targets to be specified in advance. It has particularly been used for de novo detection of splice junctions and allows genome wide expression profiling of organisms with unknown genome sequence [1].

By obtaining millions of short reads from the population of interest and by mapping these reads to the reference genome, RNA-Seq produces read count data. With enough reads from a sample, it has the potential to detect and quantify biologically significant RNAs with low and moderate abundances. Before detecting biologically significant RNAs, systematic technical variations due to experimental variability need to be removed retaining effects resulting from the biological process of interest. This process is also known as normalization. Various procedures for normalization of RNA-Seq have been proposed in literature, such as transcripts parts per million [2], trimmed mean of M values [3], and quantile normalization [4]. Though these methods have been frequently used, no comparative analysis has been presented so far.

Previous methods for identification of differential expressed genes include Bloom et al. [5] who identified differential expression by taking log ratio of the transcript counts; Hoen et al. [6] used a Student's * t*-test and alternatively also applied a Bayesian model of Vêncio et al. [7]. Marioni et al. [8] and Bullard et al. [4] suggested to use Poisson model (and Fisher's exact test, or a likelihood ratio test as an approximation to it) to test for differential expression. Recently published methods, EdgeR [9] and DESeq [10] use a Negative Binomial distribution to test for differential expression as it allows for over dispersion. We also propose two statistical methods for inferring differential expression for RNA-Seq data. They are likelihood ratio method and Bayesian method. The methods are generic and can be applied to data with or without replication.

Methods for normalization, differential expression, along with the details of the dataset used to test the performance of our methods are detailed in the next section. Results along with a systematic comparison are presented on three real datasets and we conclude with a brief discussion. 

## 2. Material and Methods 

### 2.1. Data

Datasets used to test the performance of our methods.

**Dataset 1.**

Marioni et al. [8] conducted RNA-Seq experiment with liver and kidney of a single human male using Illumina Genome Analyzer sequencing platform. Each tissue was sequenced in seven lanes, split across two runs of the machine and two different cDNA concentrations (1.5 pM, 3 pM). For this work, we only use data sequenced at 3 pM concentration (five lanes for each sample) and 17708 Ensembl transcripts that mapped with the array probes.

**Dataset 2.**

Vaz et al. [11] profiled miRNA expression from the normal peripheral blood mononuclear cells from two different individuals and cancer cells of myeloid lineage, K562 (chronic myelocytic leukemia) and HL60 (acute promyelocytic leukemia) using Solexa technology.

**Dataset 3.**

Mastrokolias et al. [12] analyzed 6 globin reduced with 6 nonreduced human whole blood RNA samples using a tag sequencing method on the Illumina high-throughput sequencing platform.

## 3. Normalization

 Normalization is a procedure to remove nonbiological influence on biological data and to make data comparable across experiments, runs, and lanes. Various normalization procedures have been proposed in literature for RNA-Seq and here we evaluate three different normalization methods: (1) transcripts parts per million, (2) trimmed mean of M values, (3) quantile normalization. At present, Transcripts parts per million (TPM) is a standard procedure to normalize RNA-Seq data. Using this method, number of reads of a transcript/sequence are divided by the total clone count of the sample and multiplied by 10^6^. Resulting normalized data is reported as reads (or transcripts) per million for each sample. One of the major problems with RNA-Seq data is that while the total number of reads for a sample is known, the composition of the RNA population is unknown. Thus, TPM normalization method has its limitations for datasets with marked different RNA composition. Trimmed mean of M values (TMM) normalization has been suggested to remove RNA compositional bias as TMM equates the overall expression levels of genes between samples by estimation of relative RNA production levels or scale factors. Another method in use is quantile normalization which has previously been applied for microarrays. In quantile normalization, the distribution of read counts in each lane is matched to a reference distribution defined in terms of median counts across sorted lanes. 

## 4. Differential Expression

 We propose two methods for inferring differential expression across two biological conditions with technical replicates, each of which yields one test statistics per gene: (i) likelihood ratio method (LRM) (Casella and Berger [13]), (ii) bayesian method (BM), an extension of technique due to Audic and Claverie [14] for more than 2 replicates within a condition. Let *x*_*j*_ denotes the observed number of reads mapped to a gene in replicate *j*(*j* = 1,2,…*m*) under condition-1 and let *y*_*j*_ denotes the observed number of reads mapped to a gene in replicate *j*(*j* = 1,2,…*n*) for condition-2. Since the number of reads mapped to a gene represents a small (less than 5%) fraction of the total number of reads obtained after sequencing, we assume *x*_*j*_ and *y*_*j*_ to follow independent Poisson distribution with different parameters. Methods are detailed for a gene and the same need to be applied for all genes. 

### 4.1. Likelihood Ratio Method

 For condition-1, *x*_*j*_ follows Poisson distribution with parameters *λ*_*j*_, *j* = 1,2,…, *m* with probability mass function as

(1) $$\begin{array}{c} p\left(x_{j}\right)=\frac{e^{-\lambda_{j}}\lambda_{j}^{x_{j}}}{x_{j}!},\;j=1,2,\ldots ,m, \end{array}$$

where *λ*_*j*_ denotes the true expression level of gene in replicate *j*. As *x*_*j*_'s occur independently, the likelihood function of *x*_1_, *x*_2_,…*x*_*m*_ is given by

(2) $$\begin{array}{c} L=L\left(\lambda_{1},\lambda_{2},\ldots ,\lambda_{m}\;|\;x_{1},x_{2},\ldots ,x_{m}\right)=\prod_{j=1}^{m}\frac{e^{-\lambda_{j}}\lambda_{j}^{x_{j}}}{x_{j}!}. \end{array}$$

To identify genes with similar read count across replicates, we test the null hypothesis *H*_0_ : *λ*_1_ = *λ*_2_ = ⋯ = *λ*_*m*_ = (say, *λ*) against the alternative *H*_1_ : *λ*_*i*_ ≠ *λ*_*j*_ for some *i* ≠ *j*. Under *H*_0_, the maximum likelihood estimate (MLE) of *λ* is given by

(3) $$\begin{array}{c} \hat{\lambda }=\frac{\sum_{j=1}^{m}x_{j}}{m}=\frac{x}{m}, \end{array}$$

where *x* = ∑_*j*=1_^*m*^ *x*_*j*_ and under *H*_1_, the MLE of *λ*_*j*_ is given by

(4) $$\begin{array}{c} {\hat{\lambda }}_{j}=x_{j},\;j=1,2,\ldots ,m. \end{array}$$

The likelihood ratio for testing *λ*_1_ = *λ*_2_ = ⋯ = *λ*_*m*_ = (say, *λ*) for condition-1 is given by

(5) Λ1=sup H0 Lsup H1 L=(x/m)x∏j=1m(xj)xj=(x)xmx∏j=1m(xj)xj.

Similarly, for condition-2, *y*_*j*_ follows Poisson distribution with parameters *μ*_*j*_, *j* = 1,2,…, *n*. As derived above, the likelihood ratio for testing *μ*_1_ = *μ*_2_ = ⋯, *μ*_*n*_ = (say, *μ*) for condition-2 is given by

(6) $$\begin{array}{c} \Lambda_{2}=\frac{{\left(y\right)}^{y}}{n^{y}\prod_{j=1}^{n}{\left(y_{j}\right)}^{y_{j}}}, \end{array}$$

where *y* = ∑_*j*=1_^*n*^ *y*_*j*_. For identifying differentially expressed genes across the two conditions, for a gene, define *x* = ∑_*j*=1_^*m*^ *x*_*j*_ and *y* = ∑_*j*=1_^*n*^ *y*_*j*_ to be independent Poisson random variables with parameters *mλ* and *nμ*, respectively, and test if *λ*≠*μ*. The joint likelihood of the two conditions is given as

(7) L=L(λ,μ ∣ x1,x2,…,xm;y1,y2,…,yn)=∏j=1me−λλxjxj!·∏j=1ne−μμyjyj!,

and the unconditional MLE's of *λ* and *μ* are given by *x*/*m* and *y*/*n*, respectively, MLE of *λ* under the hypothesis *λ* = *μ* is (*x* + *y*)/(*m* + *n*). The likelihood ratio for testing *λ* = *μ* is given by

(8) $$\begin{array}{c} \Lambda_{3}={\left(\frac{m}{m+n}\right)}^{x}{\left(\frac{n}{m+n}\right)}^{y}\frac{{\left(x+y\right)}^{x+y}}{x^{x}y^{y}}. \end{array}$$

We reject the null hypothesis for the small values of the statistic, Λ_3_. 

### 4.2. Bayesian Method

 Back in 1997, the method of Audic and Claverie was used to establish the probability distribution governing the occurrence of the same rare event in repeated experiments and was applied for the analysis of digital gene expression profiles. It was then described for only 2 replicates which we have attempted to extend to 3 or more replicates and apply to RNA-Seq data. As defined before, *x*_1_ represents the number of reads mapped to a gene in replicate 1 of the condition-1 and follows Poisson distribution

(9) $$\begin{array}{c} p\left(x_{1}\right)=\frac{e^{-\lambda }\lambda^{x_{1}}}{x_{1}!}, \end{array}$$

where *λ* denotes the actual number of reads mapped to the gene. Let *x*_2_ represents the number of reads mapped to a gene in replicate 2 of the condition-1. Then,

(10) $$\begin{array}{c} p\left(x_{2}\;|\;x_{1}\right)=\int_{0}^{\infty }p\left(d=\lambda \;|\;x_{1}\right)p\left(x_{2}\;|\;d=\lambda \right)d\lambda , \end{array}$$

where *p*(*d* = *λ*∣*x*_1_) in above equation is the posterior probability of *λ* given *x*_1_ occurrences of a gene in an experiment and *p*(*x*_2_∣*d* = *λ*) = *e* ^−*λ*^ *λ* ^*x*_2_^/*x*_2_! is the probability of drawing *x*_2_ observations from Poisson distribution with parameter *λ*. Using Bayes Theorem, Vêncio et al. [7] showed that,

(11) $$\begin{array}{c} p\left(x_{2}\;|\;x_{1}\right)=\frac{\left(x_{1}+x_{2}\right)!}{x_{1}!x_{2}!2^{\left(x_{1}+x_{2}+1\right)}}, \end{array}$$

where the prior distribution *p*(*d* = *λ*) is taken as uniform distribution over the interval [0, *∞*]. We extended the above results when the condition is replicated thrice and

(12) $$\begin{array}{c} p\left(x_{3}\;|\;x_{1},x_{2}\right)=\int_{0}^{\infty }p\left(d=\lambda \;|\;x_{1},x_{2}\right)p\left(x_{3}\;|\;d=\lambda \right)d\lambda . \end{array}$$

From Bayes Theorem,

(13) $$\begin{array}{c} p\left(d=\lambda \;|\;x_{1},x_{2}\right)=\frac{p\left(x_{1},x_{2}\;|\;d=\lambda \right)p\left(d=\lambda \right)}{\int_{0}^{\infty }p\left(x_{1},x_{2}\;|\;d=\lambda \right)p\left(d=\lambda \right)d\lambda }. \end{array}$$

Again, using uniform prior for *λ*, we get

(14) $$\begin{array}{c} p\left(d=\lambda \;|\;x_{1},x_{2}\right)=\frac{2^{x_{1}+x_{2}+1}e^{-2\lambda }\lambda^{x_{1}+x_{2}}}{\left(x_{1}+x_{2}\right)!}, \end{array}$$

which is a gamma random variable with scale parameter 2*λ*. This gives

(15) $$\begin{array}{c} p\left(x_{3}\;|\;x_{1},x_{2}\right)=\frac{2^{x_{1}+x_{2}+1}\left(x_{1}+x_{2}+x_{3}\right)!}{\left(x_{1}+x_{2}\right)!x_{3}!3^{x_{1}+x_{2}+x_{3}+1}}. \end{array}$$

Therefore,

(16) $$\begin{array}{c} p\left(x_{3}\;|\;x_{1},x_{2}\right)p\left(x_{2}\;|\;x_{1}\right)=\frac{\left(x_{1}+x_{2}+x_{3}\right)!}{x_{1}!x_{2}!x_{3}!3^{x_{1}+x_{2}+x_{3}+1}}. \end{array}$$

Similarly, if the condition is replicated *m* times, we consider the following probability.

(17) p~(x1,x2,…,xp,…,xm)  =p(xm ∣ x1,x2,…,xp,…,xm−1)⋯p(x3 ∣ x1,x2)   ×p(x2 ∣ x1)  =(x1+x2+⋯+xp+⋯+xm)!x1!x2!⋯xm!mx1+x2+⋯+xm+1.

In order to find genes with similar read counts within a condition, we find two numbers *a*, *b* such that

(18) $$\begin{array}{c} \sum_{x_{p}=0}^{a}\tilde{p}\left(x_{1},x_{2},\ldots ,x_{p},\ldots ,x_{m}\right)=\alpha ,\\ \sum_{x_{p}=b}^{\infty }\tilde{p}\left(x_{1},x_{2},\ldots ,x_{p},\ldots ,x_{m}\right)=\alpha . \end{array}$$

Equation (18) implies that if the observation *x*_*m*_ of the *m*th replicate lies in the interval [*a*, *b*] then we conclude with probability (1 − 2*α*) that there are no systematic differences between the replicates. Similarly, the results can be derived for *n* replicates of a gene in condition-2 (i.e., *y*_*j*_, *j* = 1,2,…, *n*). For Identifying differential expression across two conditions, define *x*_1_ = ∑_*j*=1_^*m*^ *x*_*j*_, *y*_1_ = ∑_*j*=1_^*n*^ *y*_*j*_ to be independent Poisson random variables with parameters *mλ* and *nμ*, respectively, and use (11). Under the Bayesian method, we can only identify genes that are different across two conditions if the number of replicates for the two conditions are the same (i.e., *m* = *n*). 

## 5. Results

### 5.1. Assessing Technical Variability Using Likelihood Ratio Method

**Figure 1:**
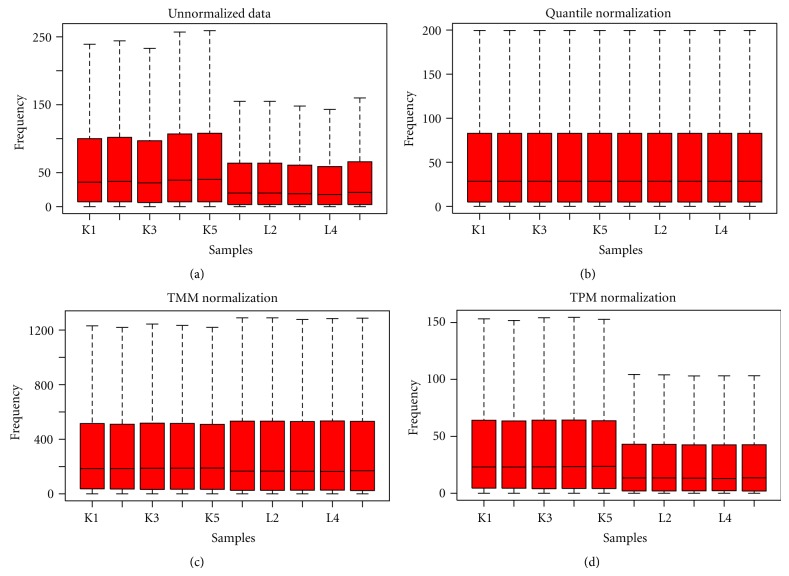
Boxplots of data from five replicates of liver and five replicates of kidney tissue: (a) Unnormalized, (b) Quantile normalized, (c) TMM normalized, and (d) TPM normalized.

 We assessed the variability within technical replicates using Dataset 1 which comprises of liver and kidney tissue, each with five technical replicates and 17708 ENSEMBL transcripts. Boxplots of unnormalized data from both liver and kidney samples are shown in Figure 1(a). Variability within replicates and also across the two tissues can be clearly seen. Kidney being more variable was considered for further analysis.

We evaluate this variability statistically using a likelihood ratio method detailed in the previous section. The analysis was performed at 1%, 2.5%, 5%, and 10% levels while considering two, three, four, and five replicates on the un-normalized data from kidney. As shown in Table 1, there is a decrease in the percentage of genes with similar counts as the number of replicates increases, which is expected; however, the decreases is only marginal. The percentage of genes with similar counts also decrease with the increase in the levels. Thus, Dataset 1 is highly reproducible with few systematic differences among the replicates.

### 5.2. Assessing the Impact of Normalization Using Likelihood Ratio Method

**Figure 2:**
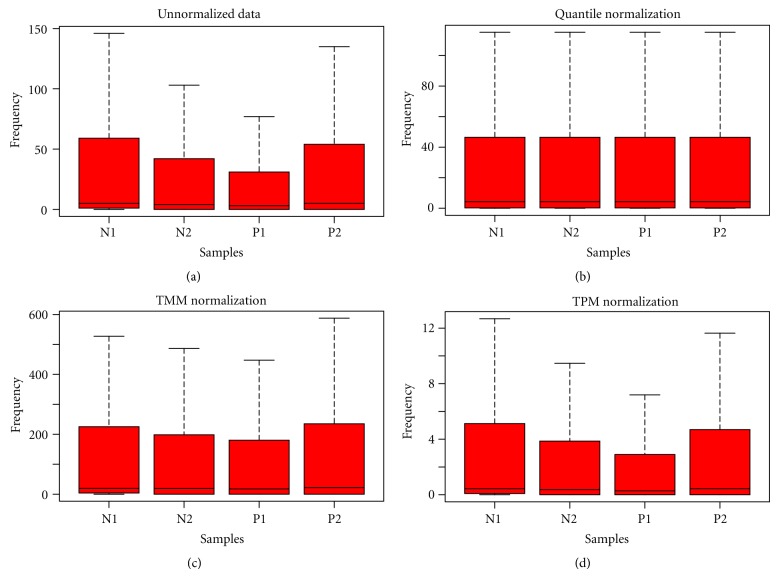
Boxplots of data from two replicates of Normal and two replicates of HL60: (a) Unnormalized, (b) Quantile normalized, (c) TMM normalized, and (d) TPM normalized.

**Figure 3:**
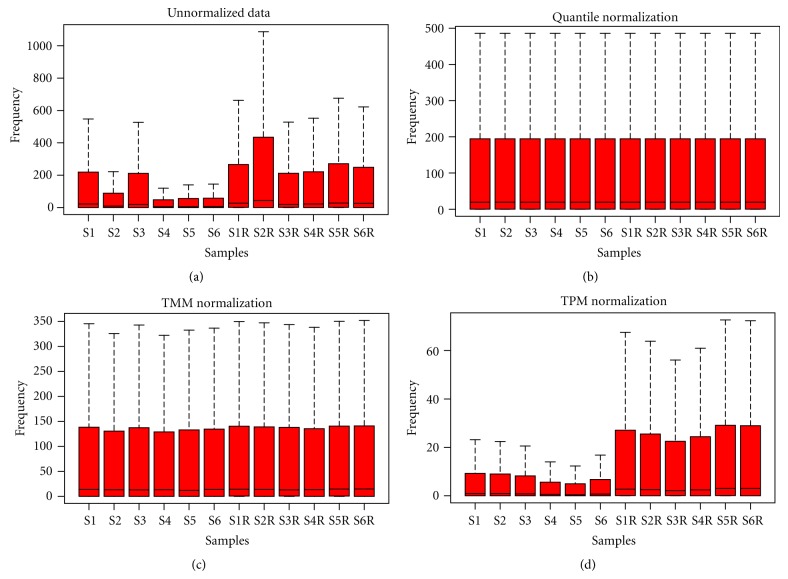
Boxplots of data from six globin reduced with 6 nonreduced human whole blood RNA samples: (a) Unnormalized, (b) Quantile normalized, (c) TMM normalized, and (d) TPM normalized.

 We assess the impact of all three normalization methods using the likelihood ratio method at 1%, 2.5%, 5%, and 10% levels. We used data from liver tissue with five replicates without normalization, with TMM, Quantile, and TPM normalization. It can be seen from Table 2 that the percentage of genes with similar counts increased after TMM and Quantile normalization and, thus, reduction in variability after normalization. A gain of 2% is achieved after TMM or Quantile normalization while the performance of TPM normalization was found to be poor. Similar results were obtained on other two datasets. Figures 1, 2, and 3 represents boxplots of un-normalized, normalized after TPM, TMM and Quantile for Datasets 1, 2, and 3, respectively.

### 5.3. Comparison of Differential Expression Statistics

 We compared the two proposed methods for inferring differentially expressed (DE) genes: Likelihood ratio method and Bayesian method on Datasets 2 and 3. We used the quantile normalized data from these datasets.

For comparison between any two biological conditions, the read count values from the conditions can be categorized under three categories. (1) When both conditions have zero count. In this situation, nothing can be said about differential expression between the two conditions. (2) When one sample has zero or low counts and a reasonable count in the other. This is an interesting biological phenomena where a gene is not expressed in one of the conditions. (3) When both the conditions have reasonable count. We shall evaluate the performance of our methods based on second and third category.

For the quantile normalized Normal versus HL60 data (Dataset 2), 19 miRNAs are absent in either of the two samples and present with a reasonable count for the other and 155 miRNAs were present with read count of at least 5 in both the samples. Using the likelihood ratio method at 1% level of significance, all 19 miRNAs absent in either of the two conditions were identified as DE and out of the 155 miRNAs, 57 were identified as DE. Using the Bayesian method at 1% level of significance, miRNAs absent in either of the two conditions were also identified as DE and out of the 155 miRNAs, 58 were identified as DE. Nearly same miRNAs, except one, were identified as DE using both the methods. We also analyzed this dataset using DESeq and EdgeR and they did not identify miRNAs absent in one of the two conditions. Of the 155 miRNAs, DESeq identified 3 miRNAs as DE with *P* value 0.01 and EdgeR identified 4 miRNAs as DE with *P* value 0.01. Similar analysis was performed for Normal versus K562 and globin reduced versus nonreduced samples. See Additional file 1, 2, and 3 in supplementary material available online at doi:10.5402/2012/817508 for detailed analysis and Table 3 for a systematic comparison between methods for all three datasets.

From Additional file 1 in supplementary material, it is clear that likelihood ratio method and Bayesian method give very similar results for Normal versus HL60 and Normal versus K562 datasets (Dataset 2). Both methods identified all miRNAs previously identified as differentially expressed in Vaz et al. [11]. However, DESeq and EdgeR could not identify most of the DE miRNAs reported in Vaz et al. [11]. Few miRNAs experimentally verified using RNase protection assay (RPA) and real-time RT-PCR in Vaz et al. [11] (i.e., miR-16, 22, 27a, 192, and let-7g) were identified with high fold in our analysis. In addition, we also identified differential expression of miR-181a family of HL60, previously reported in [15].

**Figure 4:**
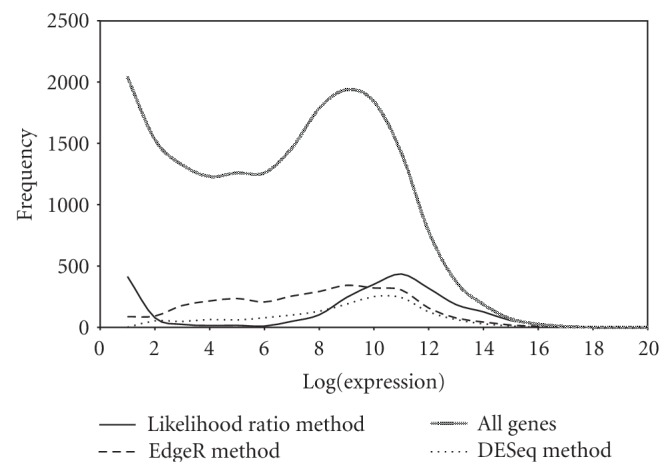
Distributions of expression strengths of all genes and significant gene list from likelihood ratio method, EdgeR and DESeq for Dataset 3.

For globin reduced versus non-reduced data (Dataset 3), likelihood method reports 2513 significant genes at 1% level of significance, Bayesian method reports 2344 at 1% level of significance, DESeq reports 1505 with *P* value 0.01 and EdgeR reports 2987 genes with *P* value 0.01. From these numbers alone, it is difficult to comment on the performance of any method.  Figure 4 demonstrates the distribution of expression strength of the significant gene list obtained from likelihood ratio method, DESeq, EdgeR, and all genes. One would expect the distribution of the significant gene lists to roughly follow the expression strength distribution for all genes. For likelihood ratio method and DESeq, this is true but not for EdgeR. EdgeR seems to be identifying genes from all expression strengths and thus not reflection biolog but the rigidity of its error models. Few genes experimentally verified in Mastrokolias et al. [12] using qPCR (i.e., CXorf25, HBA1, HBA2, HBD, HBB) were obtained with high fold values in our analysis. See additional file 3 in supplementary material for analysis.

Table 4 shows how a confidence interval was evaluated in Bayesian method for quantile normalized Normal-HL60 data. Hsa-let-7g has a read count of 15117 in Normal (condition-1) and 6236 in HL60 (condition-2). Using (18), for one replicate, we estimated the lower and upper bound of the confidence interval around Normal as 1386 and 1644. Read count of 6236 for hsa-let-7g in HL60 lies well outside the estimated confidence interval (1386, 1644). Thus, the read count in Normal and HL60 are significantly different and reported in Table 3 as T(i.e. true). Similar deductions can be made for others.

## 6. Discussions

 We assessed three different types of normalizations and showed that though Illumina data is highly replicable before normalization, normalization further reduces the technical variability, likelihood ratio method was used to statistically evaluate variation across replicates. We also presented two methods for finding differentially expressed genes for RNA-Seq data with or without replicates, likelihood ratio method is a general method that does not impose any restriction on the equality of the number of replicates across the two conditions. Bayesian method on the other hand can only be applied if there is equality on the number of replicates for the two conditions being compared. The performance of both the methods was compared to DESeq, EdgeR. For small RNA dataset, likelihood ratio method and Bayesian method perform similarly but better than EdgeR and DESeq. For Dataset 3, the distribution of the significant gene lists from likelihood ratio method and DESeq roughly follows the expression strength distribution for all genes. However, this was not true for EdgeR.

For both likelihood ratio method and Bayesian method, we assume that the underlying distribution for observed number of reads to be Poisson. Poisson distribution is intuitively appealing and mathematically easy to handle but with a limitation that the mean and variance of Poisson random variable are the same. To avoid this, authors generally assume negative binomial distribution instead of Poisson. However, the efficiency of the proposed methods in identifying differentially expressed genes, their mathematical convenience, and simplicity should make these methods extremely useful.

## Supplementary Material

Supplementary Table 1 presents the differential expression analysis for Normal vs HL60 data using Likelihood ratio method, Bayesian method, DESeq and EdgeR.
Supplementary Table 2 presents the differential expression analysis for Normal vs K562 data using Likelihood ratio method, Bayesian method, DESeq and EdgeR.
Supplementary Table 3 presents the differential expression analysis for six globin reduced vs six non reduced samples using Likelihood ratio method, Bayesian method, DESeq and EdgeR.

## Figures and Tables

**Table 1:** Assessing variability across replicates using the likelihood ratio test on 17708 genes.

No. of replicates				Percentage (number) of genes with similar count at different levels
	1%	2.5%	5%	10%
2	98.8 (17506)	97.5 (17282)	94.7 (16782)	89.2 (15809)
3	98.4 (17425)	96.6 (17111)	93.9 (16637)	88.5 (15674)
4	97.1 (17202)	94.8 (16795)	91.5 (16209)	85.8 (15197)
5	96.2 (17037)	93.5 (16563)	90.1 (15970)	84.0 (14876)

**Table 2:** Assessing variability across replicates before and after normalization using the likelihood ratio method on 17708 genes.

Type of normalization	Percentage (number) of genes with similar count at different levels			
	1%	2.5%	5%	10%
No normalization	97.6 (17294)	95.7 (16956)	93.1 (16501)	87.5 (15496)
TMM	99.0 (17540)	97.5 (17272)	95.3 (16887)	90.3 (16007)
Quantile	99.0 (17540)	97.6 (17291)	95.2 (16870)	90.3 (16002)
TPM	86.50 (15318)	86.31 (15284)	85.90 (15212)	84.50 (14964)

**Table 3:** Comparison of methods on different datasets.

Samples	Method	Number of genes present in only one samples	Number of genes present in both samples
Normal versus HL60	likelihood ratio	19	57
	Bayesian	19	58
	DESeq	0	3
	EdgeR	0	4

Normal versus K562	likelihood ratio	2	57
	Bayesian	2	53
	DESeq	0	3
	EdgeR	0	1

Globin reduced versus nonreduced	likelihood ratio	7	2513
	Bayesian	7	2344
	DESeq	5	1505
	EdgeR	7	2987

**Table 4:** Confidence interval estimation using the Bayesian method.

miRNA	Count in normal	Count in HL60	Confidence interval	Differentially expressed
Hsa-let-7g	15117	6236	[1386, 1644]	T
Hsa-miR-192	3711	2044	[3514, 3917]	T
Hsa-miR-27a	180	67	[139, 230]	T
Hsa-miR-140-5p	7	11	[1, 22]	F
Hsa-miR-30b∗	16	30	[5, 35]	F
